# Editorial Comment: Effect of pelvimetric diameters on success of surgery in patients submitted to robot-assisted perineal radical prostatectomy

**DOI:** 10.1590/S1677-5538.IBJU.2019.0413.1

**Published:** 2020-02-20

**Authors:** Gilberto J. Rodrigues, Rafael F. Coelho

**Affiliations:** 1 Instituto do Câncer do Estado de São Paulo Faculdade de Medicina da Universidade de São Paulo São PauloSP Brasil Instituto do Câncer do Estado de São Paulo, Faculdade de Medicina da Universidade de São Paulo - USP, São Paulo, SP, Brasil

## COMMENT

The outcomes of Radical prostatectomy (RP), regardless of the surgical approach, play an important role on patients' quality of life, mainly due its impact on urinary and sexual function. These outcomes are dependent on multiple factors including patient's anatomy, age, comorbidities, tumor staging, surgeon's experience, nerve sparing approach among others (1–5). Several statistical models have been published trying to predict functional and oncologic outcomes of RP based on patients' factors and perioperative parameters; these models seek to optimize preoperative counseling and patient selection for RP. However, the outcomes of RP are widely variable and conflicting results were reported with regards the importance of each factor as an independent predictor of surgical outcomes (6–11). The truth is that perioperative, functional and oncological results of RP are far more difficult to estimate, and even unknown factors may play an important role on final outcomes. Thus, in daily clinical practice, those prediction models must be cautiously interpreted and shouldn't be used as a unique tool in patients counseling or to select a specific surgical approach.

Perineal prostatectomy (RPP) was the first and oldest surgical technique described for prostate cancer treatment, progressively replaced by retropubic prostatectomy (RRP) after the introduction and standardization of the nerve-sparing technique by Walsh (12, 13). As technology and surgical techniques evolved, minimally invasive surgery emerged with laparoscopic (LRP) and robotic-assisted prostatectomy (RARP) presenting shorter length of stay, minimal blood loss and potentially better functional outcomes (14, 15). Recently, RRP was adapted to robotic-assisted platform (P-RARP) and it has been described as an option in patients with previous multiple abdominal surgeries, who presents abdominal wall defect with a mesh, obese or transplanted kidney patients, for example (16, 17). However, the real benefits of this approach in terms of surgical outcomes are yet to be proven. Despite the quite interesting findings described by Yenice at al. (18) in the current study, correlating pelvimetric measurements and operative time (but not with positive surgical margins), this findings happens to be just one more of those inconsistent predictive models with controversial results compared to other series (19, 20) and must have minimal impact on the final decision.
